# [Expression of Concern] Novel insights into the role of HSP90 in cytoprotection of H_2_S against chemical hypoxia-induced injury in H9c2 cardiac myocytes

**DOI:** 10.3892/ijmm.2025.5618

**Published:** 2025-08-28

**Authors:** Zhanli Yang, Chuntao Yang, Liangcan Xiao, Xinxue Liao, Aiping Lan, Xiuyu Wang, Ruixian Guo, Peixi Chen, Chengheng Hu, Jianqiang Feng

Int J Mol Med 28: 397-403, 2011; DOI: 10.3892/ijmm.2011.682

Following the publication of this paper, it was drawn to the Editor's attention by a concerned reader that the photos shown in Fig. 5B and D were apparently matching images, such that data which were intended to have shown the results of differently performed mitochondrial protection experiments appeared to have been derived from the same original source. The authors were contacted by the Editorial Office to offer an explanation for the apparent anomaly in the presentation of the data in this paper, although up to this time, no response from them has been forthcoming. Owing to the fact that the Editorial Office has been made aware of potential issues surrounding the scientific integrity of this paper, we are issuing an Expression of Concern to notify readers of this potential problem while the Editorial Office continues to investigate this matter further.

